# Does Electromagnetic Pollution in the ART Laboratory Affect Sperm Quality? A Cross-Sectional Observational Study

**DOI:** 10.3390/toxics13060510

**Published:** 2025-06-18

**Authors:** Giorgio Maria Baldini, Dario Lot, Daniele Ferri, Luigi Montano, Mario Valerio Tartagni, Antonio Malvasi, Antonio Simone Laganà, Mario Palumbo, Domenico Baldini, Giuseppe Trojano

**Affiliations:** 1 Obstetrics and Gynecology Unit, Department of Biomedical Sciences and Human Oncology, University of Bari “Aldo Moro”, 70121 Bari, Italy; gbaldini97@gmail.com (G.M.B.); antoniomalvasi@gmail.com (A.M.); 2 IVF Center, Momo Fertilife, 76011 Bisceglie, Italy; dariolot92@gmail.com (D.L.); danieleferrimomo@gmail.com (D.F.); 3 Unit of Andrology and Lifestyle Service in Uroandrology, ASL Salerno, 84125 Salerno, Italy; luigimontano@gmail.com; 4 Coordination Unit of the Network for Environmental and Reproductive Health (Eco-FoodFertility Project), Oliveto Citra Hospital, 84124 Salerno, Italy; 5 Clinic of Gynecology and Obstetrics, Spital Linth, Rickenstrasse 5, 8730 Uznach, Switzerland; mv.tartagni@libero.it; 6 Unit of Obstetrics and Gynecology, “Paolo Giaccone” Hospital, Department of Health Promotion, Mother and Childcare, Internal Medicine and Medical Specialties (PROMISE), University of Palermo, 90127 Palermo, Italy; antoniosimone.lagana@unipa.it; 7 Department of Public Health, School of Medicine, University of Naples “Federico II”, 80131 Naples, Italy; mpalumbomed@gmail.com; 8 Department of Maternal and Child Health, Madonna Delle Grazie Hospital, 75100 Matera, Italy; giuseppe.trojano@asmbasilicata.it

**Keywords:** electromagnetic fields, sperm motility, oxidative stress, mobile phones, Wi-Fi

## Abstract

In recent decades, exposure to electromagnetic fields (EMFs) generated by standard devices has raised concerns about possible effects on reproductive health. This cross-sectional observational study examined the impact of EMFs on sperm motility in a sample of 102 healthy males aged 20–35 years in the IVF laboratory. Semen samples were exposed to different sources of EMF for one hour, and motility was assessed immediately thereafter. The results showed a significant reduction in progressive sperm motility after exposure to EMFs generated by mobile phones and Wi-Fi repeaters in the laboratory. In contrast, other equipment showed no significant effects. The study demonstrated a statistically significant reduction in progressive sperm motility following in vitro exposure to electromagnetic fields (EMFs) emitted by mobile communication devices and wireless local area network access points. Conversely, other electromagnetic emitting devices evaluated did not elicit significant alterations in this parameter. These findings suggest a potential negative impact of specific EMF sources on semen quality, underscoring the necessity for further comprehensive research to elucidate the clinical implications and to develop potential mitigation strategies aimed at reducing risks to male reproductive health. This study discourages the introduction of mobile phones in IVF laboratories and recommends positioning Wi-Fi repeaters on the ceiling.

## 1. Introduction

In recent decades, exposure to electromagnetic fields (EMFs) has increased significantly, primarily due to the widespread use of technological devices, such as mobile phones, Wi-Fi devices, and other appliances that emit non-ionising radiation [1,2]. These devices are now ubiquitous in our daily lives, including in vitro fertilisation laboratories, where their influence on reproductive health, particularly on the quality of seminal fluid, remains a subject of scientific debate [3,4]. Among the concerns raised, one of the areas of most significant interest is the effect of EMFs on male semen quality, particularly sperm motility, a fundamental parameter for male fertility [5,6]. Several studies suggest that exposure to EMFs could impair sperm motility, reducing the ability of spermatozoa to fertilise the oocyte [7,8]. On the contrary, some research has not found significant effects [9,10], making further scientific investigation necessary. In assisted reproduction laboratories and semen analysis laboratories, the potential interference of EMFs could be amplified by the presence of multiple electronic devices, including PCs, monitors, incubators, wireless repeaters, and invertoscopes [11,12]. Some studies suggest that exposure to radiofrequency electromagnetic waves (RF-EMW) may induce oxidative stress in spermatozoa, thereby compromising their functionality [13,14]. Such oxidative stress has been associated with sperm DNA damage and reduced motility [15,16]. The mechanisms by which EMFs may affect sperm quality are still being studied, but prevailing hypotheses include oxidative damage, alterations in cellular metabolism, and possible epigenetic modifications of spermatozoa [17,18]. The interaction between EMFs and cells in the human body depends on several factors, including the intensity and duration of exposure, as well as the distance from the source of emission [19,20]. Although a large number of studies [1,2,3,4,5,6,7,8,13,14,15,16,21,22,23,24,25,26,27,28] have reported associations between exposure to EMFs and negative alterations in sperm quality, others [9,10,12,17,18,19,29,30,31]; found no significant correlations or reported conflicting results. The discrepancies observed can be attributed to methodological differences between the studies, such as the duration and intensity of exposure to EMFs, the frequency of radiation used, and the characteristics of the subjects studied. Despite the persistent uncertainty, some risk factors seem to emerge more clearly [32,33,34,35,36]. Frequent cell phone use and prolonged exposure to Wi-Fi radiation have been associated with lower sperm quality [6,7,13,15,21]. Conversely, using enclosures and maintaining a safe distance from devices could mitigate the adverse effects [6]. The global decline in semen quality observed across recent decades has significantly amplified scientific interest in the potential effects of electromagnetic fields (EMFs) on male fertility. This concerning phenomenon within the scientific community has instigated investigations into putative environmental and technological etiological factors contributing to this observed trend [37,38,39,40,41]. The pervasiveness of wireless technologies, with the consequent constant exposure to EMFs, represents a potential risk factor that deserves careful evaluation. The objective of this study is to investigate the effects of EMFs generated by devices present in the IVF laboratory on the sperm motility of apparently healthy subjects, thereby contributing to the understanding of the potential biological hazards associated with such exposure [21,22].

## 2. Materials and Methods

**Figure 1:**
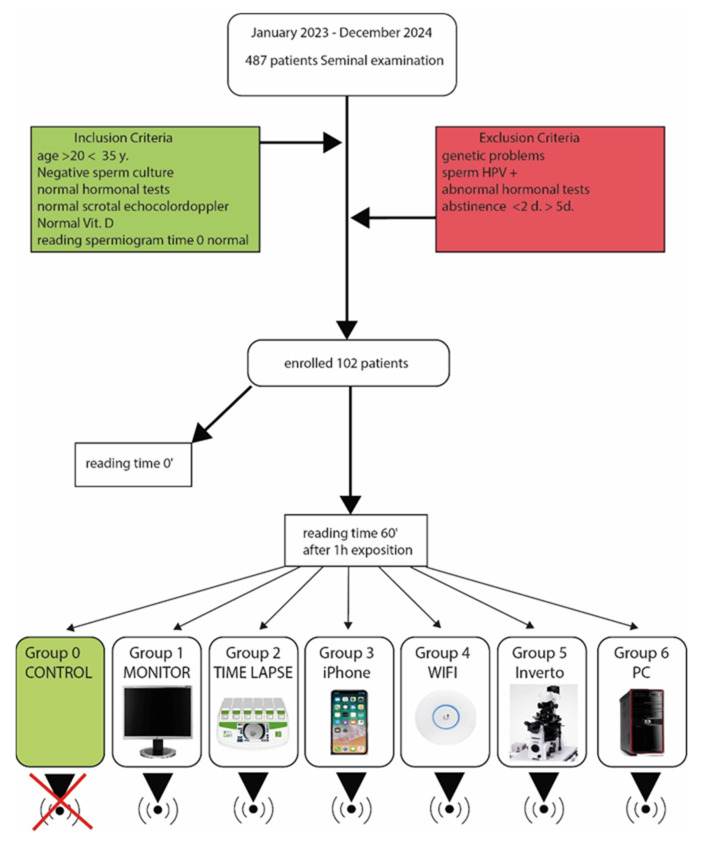
Flow chart of study design.

Study design: This is a cross-sectional observational study. Between January 2023 and December 2024, the seminal fluids of 487 males were evaluated at the MOMO FertiLIFE IVF centre in Bisceglie, Italy. From the initial number of 487 semen samples, 102 males, aged between 20 and 35, were selected. Participants were recruited voluntarily after they had administered and signed the informed consent form. The research was conducted based on the ethical principles outlined in the Declaration of Helsinki. The study received a favourable ethical assessment from the local research ethics board (CELFert reference number 11/2022). Inclusion criteria included a regular genital personal history, a normal scrotal echocolor Doppler, a negative sperm culture, and hormonal tests (FSH, LH, PRL, Testosterone, Estradiol) within normal ranges. Typical FSH values ranged from 1.5 to 12.4 mIU/mL, LH from 0.35 to 5.1 μIU/mL, prolactin from 3 to 16.5 ng/mL, testosterone from 2.27 to 9.76 ng/mL, and Estradiol from <82 pg/mL. Exclusion criteria were a positive history of genetic abnormalities, HPV positivity in semen or positive sperm culture, alteration of hormonal parameters, and abstinence for collection of less than two days and greater than 5. All semen samples were obtained after abstinence from 3 to 5 days, were collected in the morning between 09.00 and 12.00 in sterile containers and kept at room temperature for a maximum period of 30 min before being evaluated. Two evaluations were conducted: one at liquefaction (approximately 30 min after collection), which we referred to as time 0. Afterwards, the sperm sample was divided into seven aliquots; each one was exposed to an electromagnetic field generated by an electronic device, except for the one used as a control. After 60 min from the start of exposure, the semen sample was re-evaluated. The control aliquot was observed after 1 h without exposure to magnetic fields (Figure 1). The first assessment was completed with a comprehensive seminogram, while the second evaluation focused solely on motility.

### 2.1. Exposure to Electromagnetic Fields (EMF)

Since these electromagnetic fields are defined as weak, we placed the sample in a test tube at a distance of 10 cm from the device. The exposure temperature was maintained at 24 °C for all participants, with a humidity level of 60%. Considering that tubes are usually not in close contact with equipment in the laboratory, we found a distance of 10 cm to be helpful. It is essential to consider that the strengths of magnetic fields are governed by the 1/r^2^ Biot-Savart law; therefore, their intensity decreases inversely with the square of the distance. Considering that these are extremely low electromagnetic fields, a distance of more than 10 cm would significantly degrade the intensity value. The frequency affects interference and signal transmission capacity more significantly, while the strength of the magnetic field is primarily determined by the transmitter’s power and the distance.

### 2.2. Measuring Instruments

To measure the power intensity of the magnetic field, we used a portable instrument, model PCE-EM-29, from the PCE Group. We conducted continuous measurements of one hour for each device, and the results were expressed as the average exposure and maximum exposure values. The values were recorded in three-axis mode (X, Y, Z). This instrument enables sampling of frequencies between 50 MHz and 3.5 GHz with isotropic measurements in all three axes. The resolution of the power intensity of the instrument was 0.1 mV/m, with an absolute error of ±1.0 dB. The instrument had been calibrated within 1 year of the measurements taken.

The semen samples were divided into different groups, each exposed to a specific source of EMF. The duration of exposure for each group was 1 h, and exposure occurred under controlled conditions. The groups were as follows:

1. Group 0—Control. Samples were stored under normal conditions, without exposure to EMF, at time 0 and time 60’ to ensure that any variations were solely due to exposure to electromagnetic fields.
2. Group 1—Display (HP Monitor, HP Enterprise, Palo Alto, CA, USA). The samples were exposed to EMFs emitted by a 24-inch LCD monitor, placed at a distance of 10 cm from the semen samples for 1 h. The electric field power of the monitor was around 0.1 W.
3. Group 2—Time-Lapse Incubator. The samples were exposed to EMFs from a time-lapse incubator (Incubator Geri^®^, Genea Biomedx, Kent Street, Sidney, Australia) (output power: 0.5 W) at a distance of 10 cm from the sperm samples for 1 h.
4. Group 3—iPhone Cell Phone. The samples were exposed to radiation emitted by an Apple iPhone 12 mobile phone (output power: 0.1 W) (Apple Computer, Cupertino, CA, USA). The device was kept at a distance of 10 cm from the semen sample for 1 h.
5. Group 4—Ubiquiti Wi-Fi Repeater. The samples were exposed to EMFs emitted by a Ubiquiti UniFi 6 long-range Wi-Fi repeater (Ubiquiti, 685 Third Avenue, New York, NY, USA), utilising 2.4 GHz and 5 GHz Wi-Fi technology with an emission power of approximately 20 dBm (decibel milliwatts), which corresponds to approximately 100 mW (milliwatts). As in all other cases, the sample was placed at a distance of 10 cm for 1 h
6. Group 5—Nikon Invertoscope Model Ti (Nikon Instruments, Tokyo, Japan). The samples were exposed to EMFs generated by an invertoscope (semen analysis microscope) with an output power of 0.15 W, placed 10 cm away from the semen samples for 1 h.
7. Group 6—HP Pavillon TP 01 PC (HP Enterprise, Palo Alto, CA, USA). The samples were exposed to EMFs emitted by a laptop, as Wi-Fi or Bluetooth devices typically emit at very low powers (around 0.2 W), and placed at a distance of 10 cm from the semen samples for 1 h.

### 2.3. Evaluation of Sperm Motility

Sperm motility was assessed using a Nikon Eclipse E200 phase–contrast microscope (Nikon Instruments, Tokyo, Japan). The motility parameters analysed included:

- Progressive motility: percentage of spermatozoa that move in a straight line (progressive);
- Non-progressive motility;
- Immotile.

Each sample was evaluated three times by the same biologist, with the presence of another biologist serving as a witness; the results were then determined by averaging the three separate measurements.

### 2.4. Statistical Analysis

Using the G*Power software, we calculated the sample size to ensure a practical statistical evaluation. According to other studies, there is a moderate tangible effect on sperm motility after exposure to magnetic fields; therefore, we classified this as d = 0.5 (mild effect) in the Cohen classification. We have set the Alpha error probability (α) to 0.05 and power (1 − β) was set to 0.95. The allocation ratio was set to 1.00. These parameters gave a sample size of 88 patients. We analysed 102 of them. Data were analysed using SPSS software (version 25.0, IBM, Armonk, NY, USA). To assess whether there are significant differences in sperm motility (PR, NP and IM) before and after exposure to electromagnetic sources, statistically dependent tests were used, given that these are repeated measurements on the same sample. Student’s *t*-test was used after checking the normality of the data with the Kolmogorov–Smirnov test. After conducting Student’s *t*-test, we aimed to compare the electromagnetic exposure data of the various instruments and verify their impact on motility. This evaluation was performed using a Anova test.

## 3. Results

Table 1 presents data on the mean and standard deviation of the essential characteristics of the semen analysis, including days of abstinence, volume, pH, normal morphology, concentration, and total number of spermatozoa in the ejaculate. At time 0’ (after liquefaction), the progressive motility (PR), non-progressive motility (NP), and the percentage of immobile spermatozoa were evaluated. The same was performed after 1 h (time 60’).

The magnitude of the electromagnetic field intensity variations for each device is detailed in Table 2. Peak exposure levels were observed for the iPhone (1610.6 mV) and the Wi-Fi repeater (4259.2 mV), with other devices exhibiting substantially lower values. The intensity averages are clearly in favour of the iPhone (122.2 mV) and the Wi-Fi repeater (229.4 mV). All other exposure averages were much lower, with time-lapse (11.6 mV) and invertoscope (8.7 mV) being the lowest.

For a fair statistical evaluation, we wanted to assess whether the data available to us on 102 sperm fluids were usually distributed. For this reason, we used the Kolmogorov–Smirnov test. All *p*-values exceeded 0.05, thereby failing to reject the null hypothesis of normality. The distribution of the data in the QQ plots also appears to confirm normality, as the points are distributed along the diagonal line.

Table 3 reports the mean values of the three classifications of sperm motility for each exposure group, compared to the control condition. If we compare the effect of all the exposures together as the mean of the values of each and we compare them with the control group, the T student test does not show any significance. If instead we go to read the values of each exposed group, we note how those for the iPhone and for the Wi-Fi have decidedly different values. At this point, to better evaluate the various exposures among themselves, we carried out other statistical evaluations.

**Figure 2:**
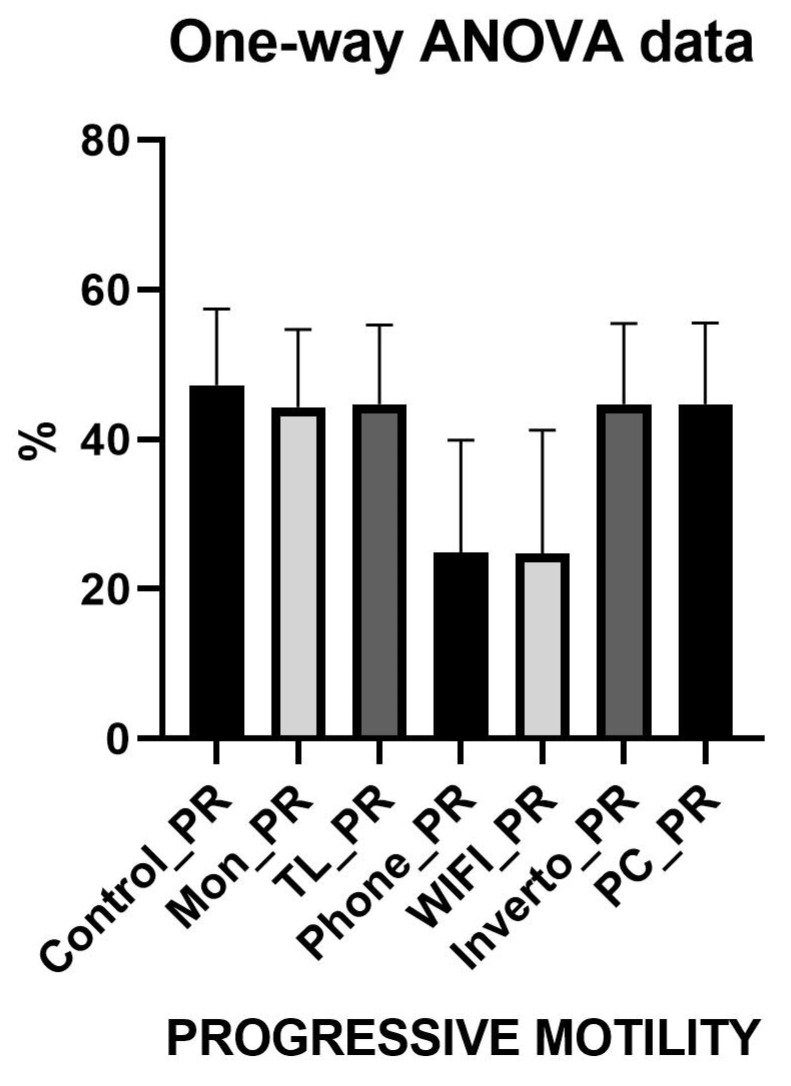
Graphic representation of Anova test of Sperm Progressive Motility.

**Figure 3:**
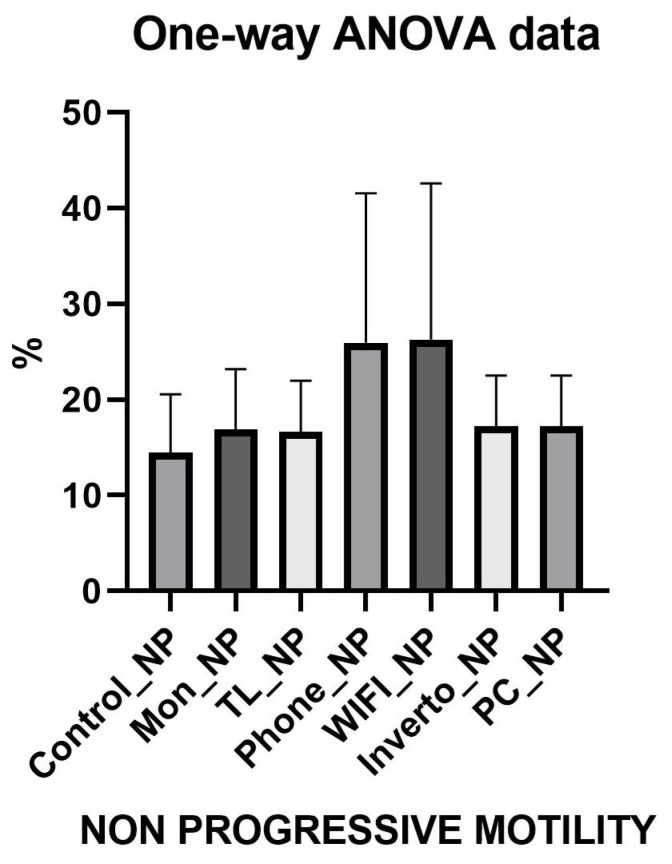
Graphic representation of Anova test of Sperm Not Progressive Motility.

**Figure 4:**
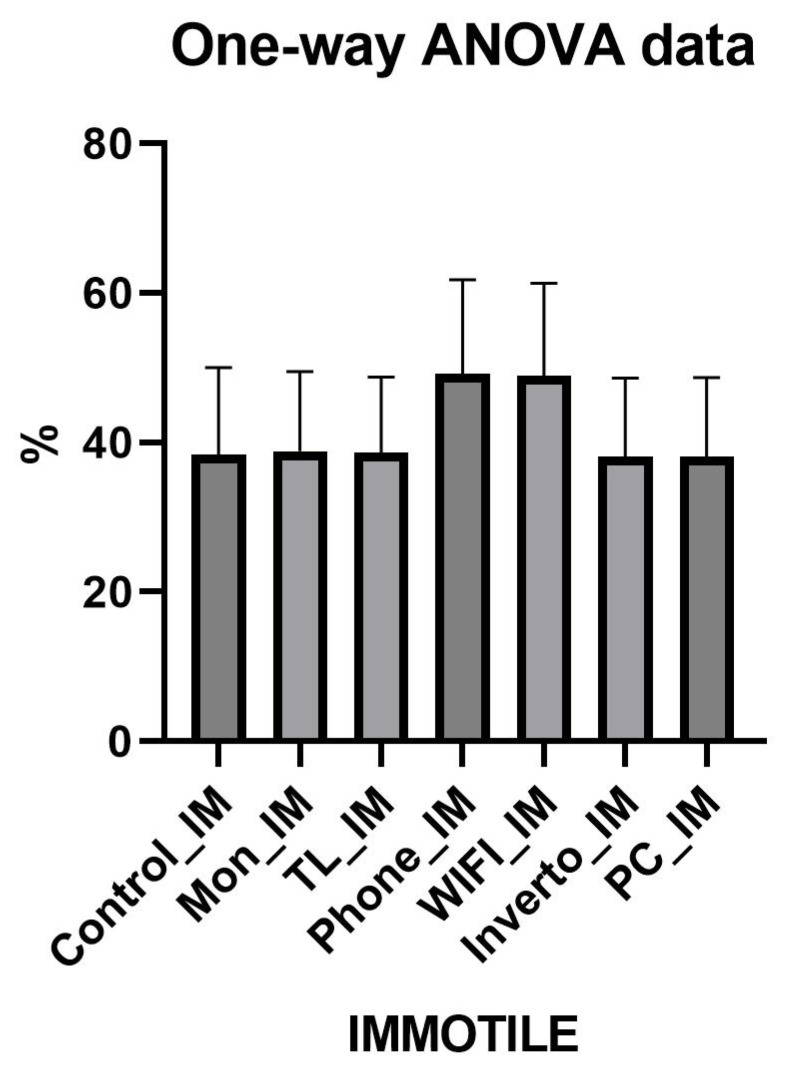
Graphic representation of Anova test of Sperm Immotile.

The comparative analysis of the three sperm motility categories (progressive, non-progressive, and immotile) was performed with the ANOVA test and revealed distinct effects depending on the environmental exposure conditions (Figure 2, Figure 3 and Figure 4). For detailed values refer to Table S1 in Supplementary material file. Results show that exposure to devices such as smartphones (Phone) and Wi-Fi networks significantly reduces progressive motility compared to controls (monitor, light towers, inverter, and PC), with an average difference of approximately 19.5 percentage points (*p* < 0.0001). This suggests that these conditions negatively impact the sperm’s ability to move effectively and directionally, a critical parameter for fertilisation. For non-progressive motility, a significant increase was observed in groups exposed to smartphones and Wi-Fi compared to controls, with an average increase of about 9 percentage points (*p* < 0.0001). This shift indicates a movement from effective progressive motility toward more limited and ineffective movement, further compromising sperm functionality. Similarly, immotile spermatozoa significantly increased in the Phone and Wi-Fi exposure groups, with an approximate 10 percentage point rise compared to control groups (*p* < 0.0001). The increased proportion of immotile sperm suggests a detrimental effect of radiofrequency and electromagnetic fields exposure on sperm viability and cellular integrity. No significant differences were found among control groups (monitor, light tower, inverter, PC) in any motility category, supporting the hypothesis that specific exposures such as smartphones and Wi-Fi have a distinct negative impact on sperm motility. These findings indicate a selective harmful effect of certain environmental exposures, presumably related to electromagnetic and radiofrequency emissions, which decrease the fraction of sperm capable of effective progressive movement and increase the non-progressive and immotile fractions, potentially compromising male fertility.

## 4. Discussion

Our study’s results suggest that exposure to electromagnetic fields (EMF) from IVF laboratory equipment did not significantly impact the overall sperm motility of the examined population. However, upon closer examination, the progressive motility (PM) of spermatozoa exposed to EMFs from mobile phones and Wi-Fi repeaters is statistically lower than in the other exposure groups. This result aligns with previous studies that have demonstrated adverse effects of EMFs on human semen quality [1,4,5,8,21,22,23,24,25,26,27,28,32,33,34,35,36,42,43,44,45,46,47]. However, some research has reported conflicting results, suggesting that the effect of EMFs may depend on variables such as the duration of exposure, radiation frequency, and the biological characteristics of the subjects examined [9,10,29,30,31,37,38,39,40,41]. It is crucial to consider the inverse square law relationship between electromagnetic field intensity and distance, underscoring the critical influence of exposure distance on the observed outcomes [48,49]. Several investigations have posited a more pronounced impact of mobile phone EMF exposure on sperm parameters [50,51]. For instance, Agarwal et al. (2009) demonstrated that exposure to mobile phone-generated EMFs elevates the production of reactive oxygen species (ROS), leading to oxidative damage and a consequent reduction in sperm motility [4]. Concordant findings were reported by De Iuliis et al. (2009), who observed an increase in oxidative stress and sperm DNA fragmentation following exposure to EMFs emitted by mobile communication devices [5]. Conversely, Avendano et al. (2012) documented a decrease in sperm motility upon exposure to Wi-Fi signals, albeit to a lesser degree than that observed with mobile phone EMFs [13]. These discrepancies in reported findings can be attributed to variations in methodological parameters, including the distance from the electromagnetic emission source, the intensity of the electromagnetic field, and the duration of exposure. In our study, the samples were exposed at a distance of only 10 cm from the radiation source, representing a relatively close exposure condition compared to the typical use of the devices. Nevertheless, as electromagnetic fields are inversely proportional to the square of the distance, the actual effects on the human body may be less marked than what has been observed in vitro [12,14,15]. A possible biological mechanism to explain the negative impact of EMFs on sperm motility is the induction of oxidative stress. EMFs can stimulate the production of ROS, which in turn can damage cell membranes and sperm DNA, impairing their functionality [1,4,5,15,16,22,25,33,43,51]. The accumulation of reactive oxygen species (ROS) can alter mitochondrial function, reducing ATP production and, consequently, the energy available for sperm motility [6,7,23,34,44]. In addition, some studies have suggested that EMFs can affect membrane fluidity and the ability of sperm to interact with the oocyte, thereby diminishing the probability of fertilisation [16,17,26,35,45]. The effect observed in our study is statistically significant, but its clinical significance remains to be evaluated. In addition to oxidative damage, other hypotheses include alteration of cellular metabolism and possible epigenetic modifications of spermatozoa [17,18,27]. EMFs could affect gene expression and DNA methylation, leading to changes in sperm function [36,46,47]. However, these mechanisms require further investigation to be fully understood. Large-scale epidemiological studies are necessary to determine whether real-world exposures to EMFs have a significant impact on human fertility [18,19,29,38]. It is essential to recognise that various factors, including age, lifestyle, exposure to chemicals, and genetics, influence male fertility. These could be confounding factors, so any subsequent studies should take this into account and perform a multivariate regressive analysis in consideration of this. Therefore, the effect of EMFs may be just one of many factors contributing to the decline in sperm quality observed in recent decades [38,47]. Sperm motility is a fundamental parameter for male fertility, and a reduction in sperm can affect the ability of spermatozoa to reach and fertilise oocytes [19,28,37]. However, chronic exposure to lower levels of EMF, such as those experienced daily, may have less pronounced effects than those observed in vitro.

### 4.1. Limitations of the Study

One of the most significant limitations concerns the sample size. Although it has been obtained through appropriate testing, it is evident that a larger sample size could enhance the validity of the results. Another limiting factor is related to the single-centre study; in fact, the results could be linked to environmental factors that limit the evidence. Another limitation is related to the device used to measure the intensity of the magnetic field, which can read frequencies between 50 MHz and 3.5 GHz. Therefore, it was unable to evaluate the total intensity of the magnetic field of the Wi-Fi repeater, which, in addition to the classic frequency of use at 2.4 GHz, also emits at 5 GHz.

### 4.2. Implications and Future Directions

The results of our study suggest that it would be prudent to limit exposure to EMFs in sensitive settings, such as assisted reproductive technology (ART) laboratories, to decrease potential risks to sperm quality. In particular, it would be recommended to avoid the presence of mobile phones and Wi-Fi repeaters in areas where gamete manipulation is being performed. Nonetheless, further studies are necessary to confirm these findings and gain a deeper understanding of the underlying mechanisms. In the future, it will be crucial to investigate the effects of prolonged and chronic exposures, as well as to assess potential interactions between EMFs and other environmental or genetic factors that may modulate sperm response [11,12,15].

## 5. Conclusions

In conclusion, our study provides further evidence of the negative impact of certain EMFs on sperm motility, highlighting that mobile phones and Wi-Fi repeaters can significantly reduce progressive sperm motility, albeit only at very close distances. All other devices do not appear to alter sperm quality in any way after exposure. Therefore, it is recommended to avoid using mobile phones near biological materials, or better yet, not to introduce them into laboratories. Additionally, it is advisable to place Wi-Fi repeaters in areas far from the handling of biological materials. Knowing that the intensity of the magnetic field is inversely proportional to the square of the distance, the application of a Wi-Fi repeater on the ceiling should not interfere with the IVF laboratory’s processing processes or the quality of the semen used.

## Figures and Tables

**Table 1:** Values of the fundamental semen analysis.

							Time 0’			Time 60’		
	Abst. day	Vol. ml.	ph	Nor. Morp. In%	Conc. Mil/mL	Total Mil.	PR%	NP%	IM%	PR%	NP%	IM%
average	3.7	3.64	7.6	9.75%	61.79	223.65	47.2%	14.5%	38.3%	45.08%	16.4%	38.7%
SD	0.7	1.22	0.2	3.41%	30.55	142.77	10.3	6.12	11.7	9.242	5.6	9.73

Legend: Abst. = abstinence; Vol. = volume; pH = pH; Nor. Morp. = Normal Morphology; Conc. = Concentration; N. Total = Total Number; PR = progressive motility; NP = not progressive motility; IM = immotile; SD = standard deviation; Mil. = million.

**Table 2:** Maximum and average values of the electromagnetic fields of the various devices.

	M_M	M_A	TL_M	TL_A	P_M	P_A	W_M	W_A	I_M	I_A	PC_M	PC_A
I.M.F. mV	1161	209.3	294.5	11.6	**1610.6**	**295.0**	**4259.2**	**241.5**	299.2	8.7	624.1	174.2
SD	88.2	8.3	15.2	2.4	122.2	12.9	229.4	17.2	22.6	2.1	15.7	11.2

Legend. M_M = Monitor Max; M_A = Monitor average; TL_M =; P_M = Phone Max; P_A = Phone average; W_M = WIFI Max; W_A = WIFI Average; I_M = I invert Max; I_A = Invert Average; PC_M = Max PC; PC_A = PC Average; I.M.F. = Intensity magnetic field measured in millivolts; SD = standard deviation.

**Table 3:** Comparison of motilities without exposure (Group 0) with the media of sum of the other groups.

	Group 1 Monitor	Group 2 Timelapse	Group 3 iPhone	Group 4 Wifi	Group 5 Invertoscope	Group 6 PC	Average of the Exposed Groups	Group 0 Control	Group 0 vs. Average Group 1 + 2 + 3 + 4 + 5 + 6 *(Test t Student)*
PR	44%	44.7%	25%	25%	45%	45%	38.11%	45.1%	N.S.
NP	16.9%	17%	25.9%	26%	17%	17%	19.9%	16%	N.S.
IM	38.9%	39%	49%	49%	38%	38%	41.9%	39%	N.S.

Legend: PR: progressive motility; NP: not progressive motility; IM: immotile; N.S.: not significant.

## Data Availability

The datasets analysed during the current study are available from the corresponding author upon reasonable request. The data will be available for 5 years from publication.

## Supplementary Materials

The following supporting information can be downloaded at: https://www.mdpi.com/article/10.3390/toxics13060510/s1, Table S1: Anova test with Tukey’s multiple comparison test for comparative analysis of the three sperm motility categories (detailed values from Anova test).
