# Supplementary material for: Does Electromagnetic Pollution in the ART Laboratory Affect Sperm Quality? A Cross-Sectional Observational Study
Source: Toxics. 2025 Jun 18;13(6):510. doi: 10.3390/toxics13060510 (PMC12197507; doi:10.3390/toxics13060510)
Supplement: Supplementary file 1 [file toxics-13-00510-s001.zip › toxics-3667729-supplementary.pdf]

Table S1. Anova test with Tukey’s multiple comparison test for comparative analysis of the three sperm motility categories.

Tukey's multiple comparisons test	Mean Diff.	95,00% CI of diff.	Significant	Summary	Adjusted P Value	Mean 1	Mean 2	Mean Diff.
**PROGRESSIVE**								
TL_PR vs. Mon_PR	0.39	−4.697 to 5.477	No	ns	>0.9999	44.71	44.32	0.39
Phone_PR vs. Mon_PR	−19.43	−24.52 to −14.34	Yes	****	<0.0001	24.89	44.32	−19.43
WIFI_PR vs. Mon_PR	−19.57	−24.66 to −14.48	Yes	****	<0.0001	24.75	44.32	−19.57
Inverto_PR vs. Mon_PR	0.38	−4.707 to 5.467	No	ns	>0.9999	44.7	44.32	0.38
PC_PR vs. Mon_PR	0.41	−4.677 to 5.497	No	ns	>0.9999	44.73	44.32	0.41
Phone_PR vs. TL_PR	−19.82	−24.91 to −14.73	Yes	****	<0.0001	24.89	44.71	−19.82
WIFI_PR vs. TL_PR	−19.96	−25.05 to −14.87	Yes	****	<0.0001	24.75	44.71	−19.96
Inverto_PR vs. TL_PR	−0.01	−5.097 to 5.077	No	ns	>0.9999	44.7	44.71	−0.01
PC_PR vs. TL_PR	0.02	−5.067 to 5.107	No	ns	>0.9999	44.73	44.71	0.02
WIFI_PR vs. Phone_PR	−0.14	−5.227 to 4.947	No	ns	>0.9999	24.75	24.89	−0.14
Inverto_PR vs. Phone_PR	19.81	14.72 to 24.90	Yes	****	<0.0001	44.7	24.89	19.81
PC_PR vs. Phone_PR	19.84	14.75 to 24.93	Yes	****	<0.0001	44.73	24.89	19.84
Inverto_PR vs. WIFI_PR	19.95	14.86 to 25.04	Yes	****	<0.0001	44.7	24.75	19.95
PC_PR vs. WIFI_PR	19.98	14.89 to 25.07	Yes	****	<0.0001	44.73	24.75	19.98
PC_PR vs. Inverto_PR	0.03	−5.057 to 5.117	No	ns	>0.9999	44.73	44.7	0.03
**NOT PROGRESSIVE**								
Mon_NP vs. TL_NP	0.25	−3.917 to 4.417	No	ns	>0.9999	16.91	16.66	0.25
Mon_NP vs. Phone_NP	−9.01	−13.18 to −4.843	Yes	****	<0.0001	16.91	25.92	−9.01
Mon_NP vs. WIFI_NP	−9.35	−13.52 to −5.183	Yes	****	<0.0001	16.91	26.26	−9.35
Mon_NP vs. Inverto_NP	−0.29	−4.457 to 3.877	No	ns	>0.9999	16.91	17.2	−0.29
Mon_NP vs. PC_NP	−0.29	−4.457 to 3.877	No	ns	>0.9999	16.91	17.2	−0.29
TL_NP vs. Phone_NP	−9.26	−13.43 to −5.093	Yes	****	<0.0001	16.66	25.92	−9.26
TL_NP vs. WIFI_NP	−9.6	−13.77 to −5.433	Yes	****	<0.0001	16.66	26.26	−9.6
TL_NP vs. Inverto_NP	−0.54	−4.707 to 3.627	No	ns	0.9991	16.66	17.2	−0.54
TL_NP vs. PC_NP	−0.54	−4.707 to 3.627	No	ns	0.9991	16.66	17.2	−0.54
Phone_NP vs. WIFI_NP	−0.34	−4.507 to 3.827	No	ns	>0.9999	25.92	26.26	−0.34
Phone_NP vs. Inverto_NP	8.72	4.553 to 12.89	Yes	****	<0.0001	25.92	17.2	8.72
Phone_NP vs. PC_NP	8.72	4.553 to 12.89	Yes	****	<0.0001	25.92	17.2	8.72
WIFI_NP vs. Inverto_NP	9.06	4.893 to 13.23	Yes	****	<0.0001	26.26	17.2	9.06
WIFI_NP vs. PC_NP	9.06	4.893 to 13.23	Yes	****	<0.0001	26.26	17.2	9.06
Inverto_NP vs. PC_NP	0	−4.167 to 4.167	No	ns	>0.9999	17.2	17.2	0
**IMMOTILE**								
Mon_IM vs. TL_IM	0.22	−4.296 to 4.736	No	ns	>0.9999	38.85	38.63	0.22
Mon_IM vs. Phone_IM	−10.34	−14.86 to −5.824	Yes	****	<0.0001	38.85	49.19	−10.34
Mon_IM vs. WIFI_IM	−10.14	−14.66 to −5.624	Yes	****	<0.0001	38.85	48.99	−10.14
Mon_IM vs. Inverto_IM	0.75	−3.766 to 5.266	No	ns	0.997	38.85	38.1	0.75
Mon_IM vs. PC_IM	0.78	−3.736 to 5.296	No	ns	0.9964	38.85	38.07	0.78
TL_IM vs. Phone_IM	−10.56	−15.08 to −6.044	Yes	****	<0.0001	38.63	49.19	−10.56
TL_IM vs. WIFI_IM	−10.36	−14.88 to −5.844	Yes	****	<0.0001	38.63	48.99	−10.36
TL_IM vs. Inverto_IM	0.53	−3.986 to 5.046	No	ns	0.9994	38.63	38.1	0.53

TL_IM vs. PC_IM	0.56	−3.956 to 5.076	No	ns	0.9993	38.63	38.07	0.56
Phone_IM vs. WIFI_IM	0.2	−4.316 to 4.716	No	ns	>0.9999	49.19	48.99	0.2
Phone_IM vs. Inverto_IM	11.09	6.574 to 15.61	Yes	****	<0.0001	49.19	38.1	11.09
Phone_IM vs. PC_IM	11.12	6.604 to 15.64	Yes	****	<0.0001	49.19	38.07	11.12
WIFI_IM vs. Inverto_IM	10.89	6.374 to 15.41	Yes	****	<0.0001	48.99	38.1	10.89
WIFI_IM vs. PC_IM	10.92	6.404 to 15.44	Yes	****	<0.0001	48.99	38.07	10.92
Inverto_IM vs. PC_IM	0.03	−4.486 to 4.546	No	ns	>0.9999	38.1	38.07	0.03

Legend PR = progressive NP = non progressive IM = Immmotile
